# Noise Induces Biased Estimation of the Correction Gain

**DOI:** 10.1371/journal.pone.0158466

**Published:** 2016-07-27

**Authors:** Jooeun Ahn, Zhaoran Zhang, Dagmar Sternad

**Affiliations:** 1 Department of Mechanical Engineering, University of Victoria, Victoria, British Columbia, Canada; 2 Department of Bioengineering, Northeastern University, Boston, Massachusetts, United States of America; 3 Department of Biology, Electrical & Computer Engineering and Physics, Northeastern University, Boston, Massachusetts, United States of America; University of California, Merced, UNITED STATES

## Abstract

The detection of an error in the motor output and the correction in the next movement are critical components of any form of motor learning. Accordingly, a variety of iterative learning models have assumed that a fraction of the error is adjusted in the next trial. This critical fraction, the correction gain, learning rate, or feedback gain, has been frequently estimated via least-square regression of the obtained data set. Such data contain not only the inevitable noise from motor execution, but also noise from measurement. It is generally assumed that this noise averages out with large data sets and does not affect the parameter estimation. This study demonstrates that this is not the case and that in the presence of noise the conventional estimate of the correction gain has a significant bias, even with the simplest model. Furthermore, this bias does not decrease with increasing length of the data set. This study reveals this limitation of current system identification methods and proposes a new method that overcomes this limitation. We derive an analytical form of the bias from a simple regression method (Yule-Walker) and develop an improved identification method. This bias is discussed as one of other examples for how the dynamics of noise can introduce significant distortions in data analysis.

## Introduction

Error detection and correction is one of the most fundamental elements in motor learning, in fact in almost every type of learning. In engineering, the notion that the error in the current execution is used to improve the next execution is the essence of any controller design for systems that perform tasks repeatedly [1–3]. In human learning, it is universally agreed upon that the perception and correction of errors plays a significant role in the process of motor learning [4, 5], although the neural and cognitive mechanisms underlying error-based learning are not yet fully understood [6, 7]. Feedback of error, and knowledge of results or performance have long been shown to facilitate and accelerate acquisition of new motor skills [8]. Based on this understanding, motor learning can be enhanced by magnifying the perceived error [9–12]. Errors are not only instrumental during early practice, but smaller corrections also continue into later stages of skilled performance. Prior studies highlighted the critical role of error corrections during the repeated execution of goal-directed movements near the goal [13–15]. In fact, expert performance at a highly skilled level implies that any small error is readily compensated for.

While ubiquitous, it remains an open question to what degree each error is corrected for. Should a learner completely compensate for each error in the subsequent attempt, or should he/she be more conservative and only correct a smaller portion of the error? If random noise is part of the observed fluctuations, then complete error cancellation may lead to an overshoot and ultimately unstable performance. As errors become smaller with practice, should this proportion or “gain” of the correction change? With these questions, the sensitivity of error correction, the gain or learning rate, has been a key variable in several areas of research.

With the goal to shed light on error-based learning, various mathematical models have been used to quantify detection and correction of error. For example, Galea et al. used a simple deterministic model to reveal differences in the learning rate under positive and negative reward in a motor adaptation task [16]. Smith et al. introduced a learning model with error feedback on two different time scales that reproduced several features of human motor adaptation, including savings [17]. However, neither of the two models considered noise, which is inevitable and, arguably, even beneficial to motor learning [18–21]. Heeding to the critical role of random fluctuations, Scheidt et al. proposed an exponential model for learning of goal-directed reaching under random perturbations [22]. Baddeley et al. compared a series of simple models that included noise sources to find the best fit for human visuo-motor performance; results suggested that exponential weighting of recent errors may best account for the high efficacy of human performance [23]. Diedrichsen et al. also used a stochastic state-space model of trial-by-trial adaptation and suggested a dissociation between errors that guide behavioral goals and those that guide neural adaptation of internal models [24]. Several other studies used stochastic iterative models to address how error corrections are associated with a structural change of variability using skills ranging from simple reaching [13, 25] to throwing a ball [14].

The most critical parameter in all these learning models is the correction gain, learning rate or feedback gain, which determines how much of the error in the current trial is corrected in the following trial. The simplest form of an iterative learning model that includes a correction gain and noise can be described by a set of three linear equations [13]:

$$x_{i}=m_{i}+N_{i}$$ (1)

$$e_{i}=x_{i}-x_{\text{target}}$$ (2)

$$m_{i+1}=m_{i}-Be_{i}$$ (3)

Eq 1 defines the motor output, **x** at the *i*^th^ trial as a sum of the planned execution **m** and noise **N**; the latter is a random vector drawn from a zero mean distribution with a known covariance matrix. Most learning models assume that this noise comes from a Gaussian distribution. Eq 2 defines the error as the difference between the output and the target. Eq 3 models the learning process as an update of the planned execution based on the error information. The constant *B* is the correction gain, which defines the fraction of the error that is adjusted in the next trial.

Conventionally, estimation of this critical parameter *B* in a given time series of data has relied on a convenient and common mathematical tool—the method of least-squares regression. Different parameter optimization algorithms include the Gauss-Newton algorithm, gradient descent, and the Levenberg-Marquardt algorithm [26–29]. These model-based methods have been applied regardless of possible noise in the model or in the measured data. Implicitly or explicitly, these approaches relied on the assumption that the effect of noise averages out. If noise is explicitly taken into account using stochastic models, a widely-used method is the Expectation-Maximization (EM) algorithm [30]. This algorithm estimates the model parameters including the noise via maximum likelihood estimation [31, 32]. However, this method is confined to models with independent noise sources and cannot be applied to auto-regressive processes [33].

The present study closely examined the viability of least-square methods for estimating model parameters, scrutinizing the common assumption that noise averages out. As we will demonstrate, the subtle dynamics of noise significantly influence the estimates of the correction gain and produce a bias. This bias may be relatively small when the difference between initial and final motor output is by orders of magnitude larger than the noise variance, as is the case at initial stages of learning. However, when the learning process approaches steady-state, or the error is comparable with the variability of the motor output, the bias in the estimated correction gain can become significant.

We devise a new method that improves the accuracy of identification of the correction gain, for the case when motor learning is close to steady state and the error is of similar magnitude as the overall variability. Analytical approaches and numerical simulations show that the quantified bias and the adjustment by the new method are robust and are insensitive to the magnitude and the distribution of the noise.

## Methods

### A Simple Iterative Model

To exemplify the problem of least-square estimation of the correction gain and demonstrate the bias of the conventional estimate, we examined a simple model that represents the basic structure of a large number of learning models [13, 14, 23–25]. For the simplest one-dimensional case, the vector variables in Eqs 1 to 3 become scalars. Defining the origin of the coordinate as the desired motor output, or the target state (*x*_target_ = 0),

$$x_{i}=m_{i}+N_{i},$$ (4)

$$e_{i}=x_{i},$$ (5)

$$m_{i+1}=m_{i}-Be_{i},$$ (6)

where *N*_*i*_ is a random variable from a distribution with zero mean and a standard deviation of *σ*_*N*_, and *B* is assumed to satisfy 0 < *B* < 1. From Eqs 4, 5 and 6,

$$x_{i+1}=m_{i+1}+N_{i+1}=m_{i}-Be_{i}+N_{i+1}=m_{i}-Bx_{i}+N_{i+1}=x_{i}-N_{i}-Bx_{i}+N_{i+1}.$$

Therefore, the learning process can be described by a simple linear equation:

$$x_{i+1}=(1-B)x_{i}+N_{i+1}-N_{i}.$$ (7)

In this auto-regressive form, least-square regression can be used to estimate *B* from a time series of measured data.

### Testing the Validity of the Conventional Least-Square Regression

To test whether the least-square estimation extracts the real parameters with sufficient accuracy, the first step was to generate a set of time series {*x*_*k*_} using the simple model of Eq 7 with a known value of *B* and random noise samples. The lengths of the time series *n* varied from 10 to 800. To extract the correction gain and the variance of the noise, Eq 7 was analyzed using the standard regression approach. We opted to use the Levenberg-Marquardt algorithm [28] among many other alternative least-square algorithms because this algorithm is an effective numerical method for regression. It has therefore been a common choice for least-squares problems in commercialized numerical computing environments including Matlab (Mathworks Inc., Natick, MA). The Levenberg-Marquardt algorithm has also been widely applied in motor learning studies [34, 35] and for the training of neural networks [36–38].

**Fig 1:**
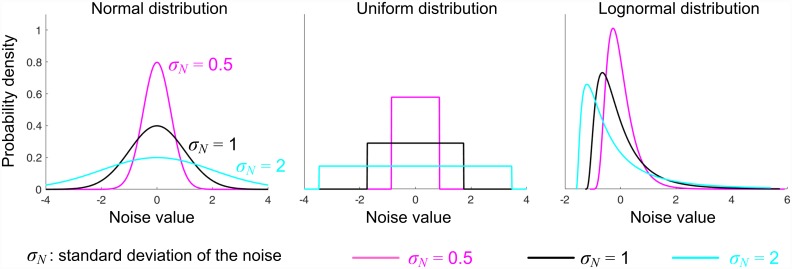
Probability density functions of the applied noise. Three types of noise distributions (normal, uniform and lognormal) with three different standard deviations were considered. For each type of distribution, the expectation value of the noise is zero, whereas the standard deviation *σ*_*N*_ varies from 0.5 to 2, resulting in different noise levels.

We generated time series with three different *B* values of 0.25, 0.50, and 0.75. As little is known about the origin and magnitude of the noise in actual data, we also investigated the effect of the noise distribution with three types of distributions—normal, uniform, and asymmetric lognormal. In addition, we simulated different magnitudes of the noise level *σ*_*N*_. The probability density functions of the added noise are demonstrated in Fig 1.

The simulation and subsequent parameter estimation were repeated 1000 times for each parameter set and time series length *n*. The difference between the true *B* and the mean of the 1000 estimated values $\hat{B}$ was evaluated for each noise distribution, noise level, and *n*. Numerical simulations and analyses were implemented in Matlab (Mathworks Inc., Natick, MA).

### Quantification of the Bias and Development of Improved Estimation: Adjusted Yule-Walker (AYW) Method

Following the demonstration of a significant bias as reported in the results, we proceeded to show that this bias can be removed if an analytical expression of the bias is obtained. To attain a closed form of the bias, we considered one of the simplest estimators, the Yule-Walker algorithm [39, 40]. Note that the Yule-Walker estimation method does not reduce the bias by itself. However, it lends itself to obtain an analytical expression of the bias due to its simple structure. The same analytical derivation might be done for the commonly used Levenberg-Marquardt algorithm, but would be more challenging and less transparent. Using the derived correction term, this bias can then be eliminated, which significantly improves the accuracy of the parameter estimation.

For any autoregressive (AR) process, the general Yule-Walker method calculates the AR parameters. The algebraic expression of the simplest AR process of order one is

$$x_{i+1}=Ax_{i}+N_{i+1},$$ (8)

where *N*_*i*_ is a random variable. The Yule-Walker equation estimates the parameter *A* as

$$A_{YW}=\frac{\sum_{i=1}^{n-1}x_{i}x_{i+1}}{\sum_{i=1}^{n-1}x_{i}{}^{2}},$$ (9)

which is identical to the least-square linear regression. Note that Eq 8, which is a first-order AR process, has a similar structure as the simple learning model in Eq 7. The assumption that the noise in each iteration is an independent random sample allowed to approximate the expected bias of the Yule-Walker estimation in a simple closed form. Using the closed form of the bias, we could then quantify the correction gain more accurately. We called the procedure the Adjusted Yule-Walker method (AYW).

**Fig 2:**
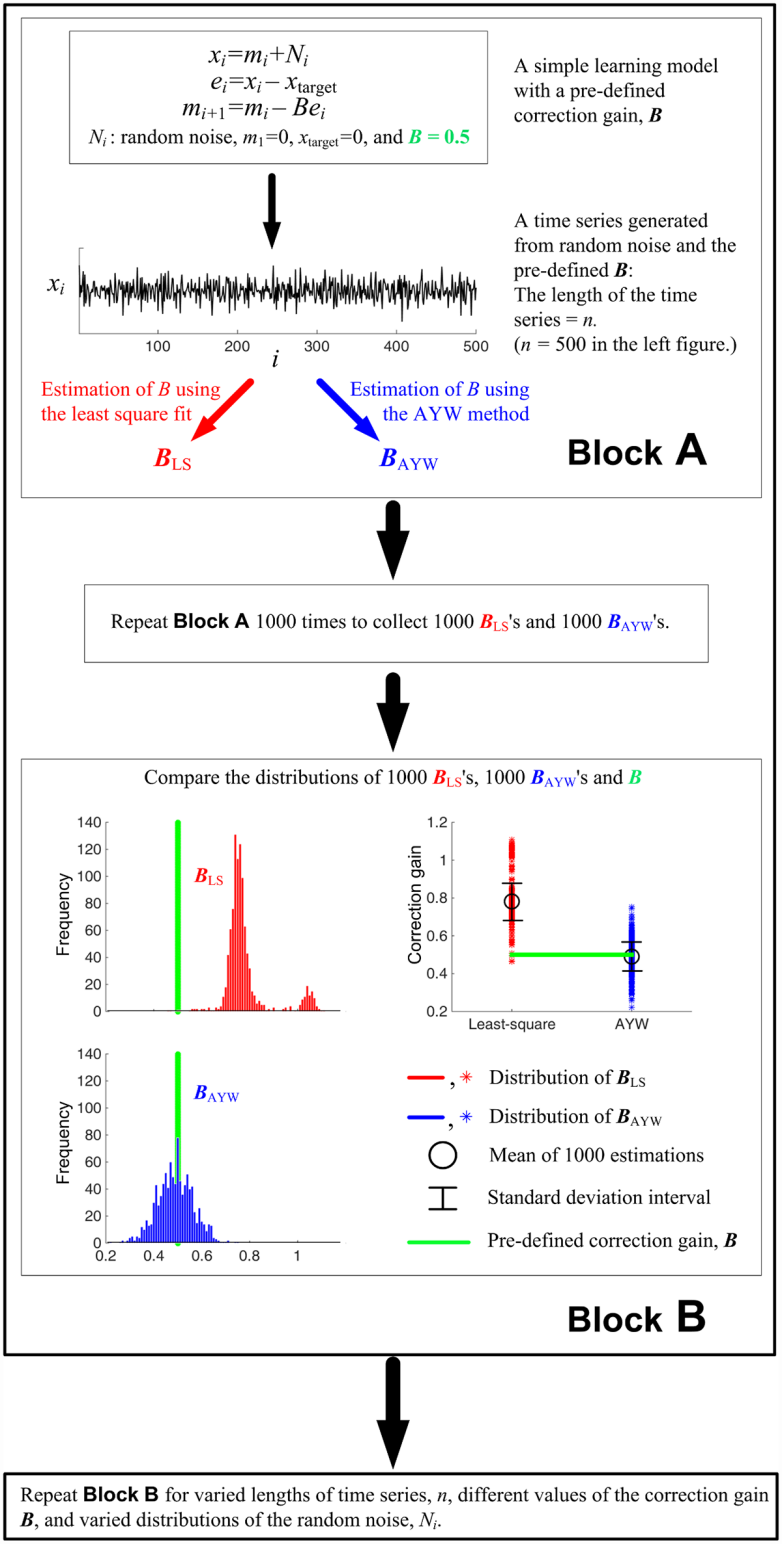
Illustration of the analysis method and a representative data set.

We verified the reliability of this AYW method in the same way as we tested the reliability of the conventional least-square method: with a fixed value of the correction gain *B*, we constructed a set of time series {*x*_*k*_} with added noise; the length of the time series *n* varied from 10 to 800. As above, the simulations and estimation were performed with three different noise distributions and three different noise levels (Fig 1). The simulation was repeated 1000 times for each *n* and each parameter set. The correction gain *B* was estimated by the AYW method, and the difference between the true *B* value and the mean of the 1000 estimated values $\hat{B}$ was evaluated for each noise distribution, noise level, and *n*. The entire procedure is overviewed in Fig 2.

## Results

### Demonstration of the Bias from Least-Square Estimation

**Fig 3:**
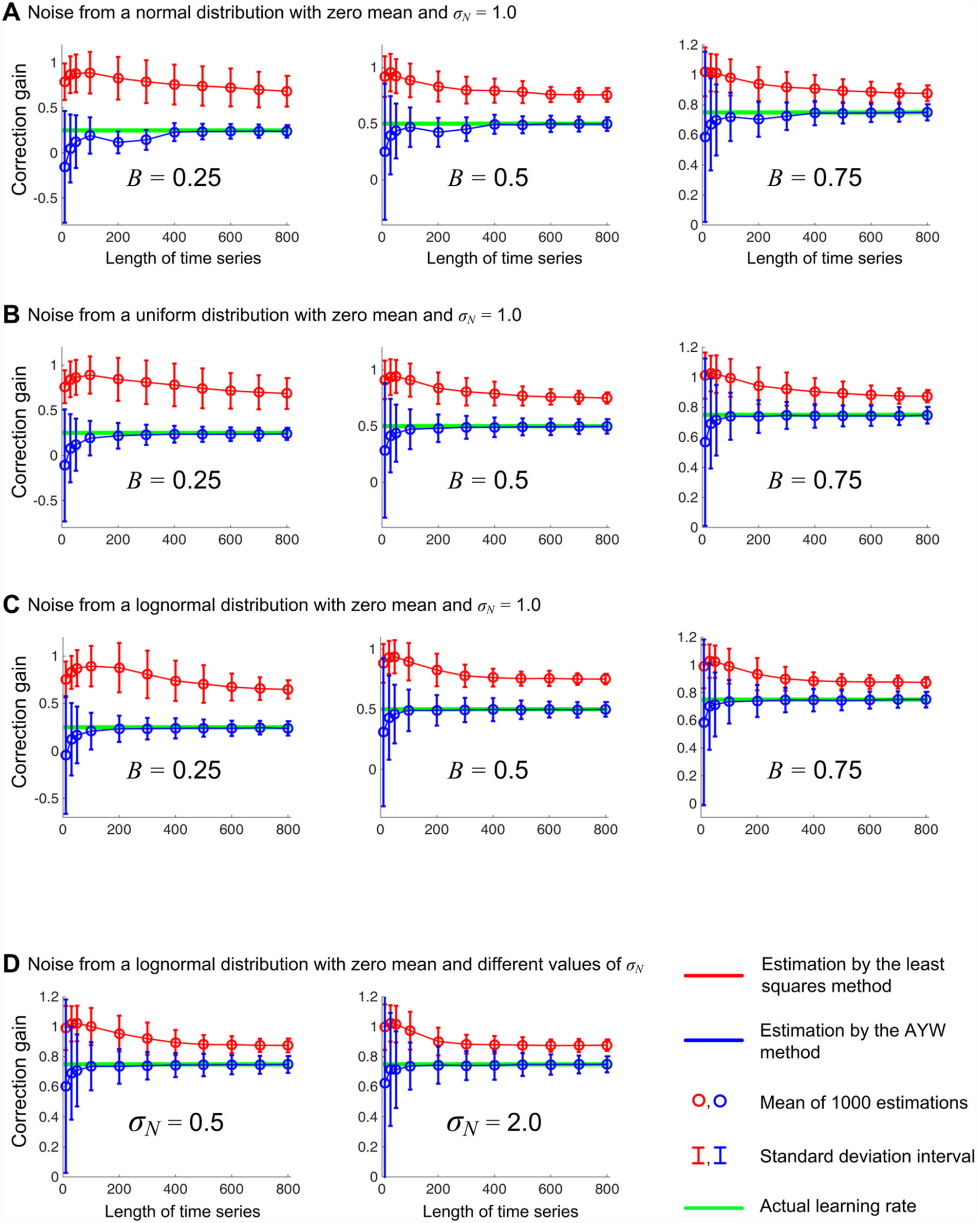
Actual and estimated correction gain *B*. Time series with actual correction gains *B* of 0.25, 0.5 and 0.75; different trial length *n* (from 10 to 800); and three distributions of noise (normal, uniform, and lognormal) were generated using Eq 7. The correction gain *B* was estimated for each time series using both the Levenberg-Marquardt least-square algorithm (red) and the Adjusted Yule-Walker (AYW) method (blue), and then compared with the actual correction gain *B* (green). The simulations were repeated 1000 times for each algorithm and each *n*. The estimation by the Levenberg-Marquardt algorithm yielded a substantial bias, whereas the AYW method significantly reduced the bias for large enough *n*. These results remained unchanged for the noise sampled from a Gaussian (A), a uniform (B), or an asymmetric lognomal (C) distribution. In addition, the bias from the Levenberg-Marquardt algorithm and the improvement by the AYW method were not affected by the magnitude of the noise level *σ*_*N*_ (D).

The conventional Levenberg-Marquardt (LM) algorithm based on the least-square method yielded a substantial bias. Fig 3 shows the *B* values for different time series length *n* estimated with the LM-algorithm (red) and the AYW method (blue) against the real *B* value (green). The estimated correction gains $\hat{B}$ had higher values than the actual correction gains *B*. Table 1 summarizes and compares the magnitude of the biases when the correction gain was estimated by the two methods. To facilitate comparison, a normalized error was calculated as $({\hat{B}}_{avg}-B)/B\times \;100\;\left(\%\right)$, where ${\hat{B}}_{avg}$ is the mean of the 1000 estimates, and *B* is the actual correction gain. Depending on the true value of *B*, the bias from the LM estimates was at 16 ~ 170% of *B*, even when the length of the time series *n* reached 800. The biases in the AYW method became less than 5% as *n* reached 800. Further, in Fig 3, the panels A to C show that the significant bias did not change much whether the noise came from a normal, uniform, or asymmetric lognormal distribution. Panel D in Fig 3 also shows that the bias was largely unaffected by the level of the noise.

**Table 1:** Normalized biases in the estimates of the correction gain *B* using the least-square methods and the Adjusted Yule-walker method.

Noise from normal distribution												
		**Trial length, *n***										
***B***	**Method**	**10**	**30**	**50**	**100**	**200**	**300**	**400**	**500**	**600**	**700**	**800**
0.25	Least-square	220	250	250	260	230	220	200	200	190	180	170
0.25	AYW	-160	-80	-50	-22	-53	-42	-7.5	-5.1	-4.3	-3.2	-4.3
0.50	Least-square	84	91	84	77	66	60	59	56	52	51	51
0.50	AYW	-50	-21	-13	-6	-15	-10	-1.1	-2.1	-0.34	-0.69	-0.81
0.75	Least-square	36	35	35	31	25	22	21	19	18	17	17
0.75	AYW	-21	-11	-6.7	-3.9	-6.2	-3.4	-0.63	-0.69	-0.53	-0.25	0.031
Noise from uniform distribution												
		**Trial length, *n***										
***B***	**Method**	**10**	**30**	**50**	**100**	**200**	**300**	**400**	**500**	**600**	**700**	**800**
0.25	Least-square	200	240	250	260	240	220	210	200	190	180	170
0.25	AYW	-140	-68	-53	-23	-13	-8.7	-6.1	-6.3	-5.9	-5.9	-4.5
0.50	Least-square	82	87	88	82	67	61	58	54	52	51	50
0.50	AYW	-43	-17	-12	-5.5	-3.8	-2.1	-2.1	-1.3	-1.3	-0.78	-0.48
0.75	Least-square	35	37	36	33	26	23	21	19	18	17	17
0.75	AYW	-24	-7.7	-4.8	-1.3	-1.5	-0.50	-0.97	-0.92	-0.82	-1.1	-0.34
Noise from lognormal distribution												
		**Trial length, *n***										
***B***	**Method**	**10**	**30**	**50**	**100**	**200**	**300**	**400**	**500**	**600**	**700**	**800**
0.25	Least-square	200	230	250	260	250	220	200	180	170	160	160
0.25	AYW	-120	-50	-33	-16	-6.6	-5.9	-4.5	-3.4	-4.7	-1.8	-4.7
0.50	Least-square	76	86	87	79	65	56	53	51	51	50	50
0.50	AYW	-38	-14	-8.0	-1.8	-1.8	-1.3	-0.61	-0.85	-0.44	-0.92	-0.16
0.75	Least-square	32	37	36	32	24	20	18	17	17	17	16
0.75	AYW	-22	-6.7	-4.8	-2.1	-1.4	-0.47	-0.61	-0.85	-0.56	0.0085	-0.083

The biases were calculated for each type of noise distribution (normal, uniform, or lognormal) and each trial length in a normalized form of $({\hat{B}}_{avg}-B)/B\times \;100\;\left(\%\right)$, where ${\hat{B}}_{avg}$ is the mean of the 1000 estimates, and *B* is the actual value of the correction gain. Note that the conventional least-square method with the Levenberg-Marquardt algorithm yielded biases of 16 ~ 170%, even with a large trial number. The biases with the AYW method became less than 5% as *n* reached 800.

### Analytical Derivation of the Bias from the Yule-Walker Equation

We analytically derived the bias in the estimates of the correction gain using the Yule-Walker equation [39, 40], one of the simplest regression methods. The notation of the one-dimensional learning process of Eq 7 is simplified by substituting (1 –*B*) with *C*,

$$x_{i+1}=Cx_{i}+N_{i+1}-N_{i},$$ (10)

where *N*_*i*_ is a random variable from a distribution with zero mean and the standard deviation of *σ*_*N*_, and *C* is between 0 and 1 because 0 < *B* < 1.

To illustrate,

$$\begin{array}{ll} x_{1} & =N_{1}\\ x_{2} & =CN_{1}+N_{2}-N_{1}=(C-1)N_{1}+N_{2}\\ x_{3} & =C(C-1)N_{1}+CN_{2}+N_{3}-N_{2}=C(C-1)N_{1}+(C-1)N_{2}+N_{3}\\ \vdots & \ddots \\ x_{n} & =C^{n-2}(C-1)N_{1}+C^{n-3}(C-1)N_{2}+C^{n-4}(C-1)N_{3}+\cdots +(C-1)N_{n-1}+N_{n} \end{array}$$ (11)

To warrant using the Yule-Walker equation, or Eq 9, we regard *N*_*i*+1_ –*N*_*i*_ in Eq 8 as another single noise term, such that the coefficient *C* can be estimated as

$$C_{YW}=\frac{\sum_{i=1}^{n-1}x_{i}x_{i+1}}{\sum_{i=1}^{n-1}x_{i}{}^{2}}.$$ (12)

From Eq 11, the expectation of *x*_*n*_ is a weighted sum of the expectation of *N*_*i*_, each of which is zero. Therefore,

$$E[x_{n}]=0.$$ (13)

By definition, *N*_*p*_ and *N*_*q*_ are independent when *p* ≠ *q*. Therefore,

$$E\left(N_{p}N_{q}\right)\;=\;0\;\text{if}\;p\neq q$$ (14)

because

$$\int_{-\infty }^{\infty }\int_{-\infty }^{\infty }N_{p}N_{q}f(N_{p})f(N_{q})dN_{p}dN_{q}=\int_{-\infty }^{\infty }N_{q}\left(\int_{-\infty }^{\infty }N_{p}f(N_{p})dN_{p}\right)dN_{q}=0,$$

where *f* is the probability density function of the noise. Note that the validity of Eq 14 does not depend on the specific property of the distribution. Eq 14 remains valid, regardless of whether the probability density function *f* is symmetric like a normal and a uniform distribution, or asymmetric like a lognormal distribution. From Eq 11, it follows that

$$\begin{array}{l} E\left[x_{n}{}^{2}\right]=E\left[{\left(C^{n-2}(C-1)N_{1}+C^{n-3}(C-1)N_{2}+C^{n-4}(C-1)N_{3}+\cdots +(C-1)N_{n-1}+N_{n}\right)}^{2}\right]\\ =E\left[C^{2(n-2)}{\left(C-1\right)}^{2}N_{1}{}^{2}+\cdots +{\left(C-1\right)}^{2}N_{n-1}{}^{2}+N_{n}{}^{2}+\text{terms with }N_{p}N_{q}\text{ (}p\neq q)\right]\;. \end{array}$$

By Eq 14, terms with *N*_*p*_ *N*_*q*_ (*p* ≠ *q*) do not contribute to the expectation. In addition, by the definition of *N*_*i*_, the expectation of $N_{i}{}^{2}$ is *σ*_*N*_^2^. Therefore,

$$E\left[x_{n}{}^{2}\right]=C^{2(n-2)}{\left(C-1\right)}^{2}\sigma_{N}{}^{2}+C^{2(n-3)}{\left(C-1\right)}^{2}\sigma_{N}{}^{2}+\cdots +{\left(C-1\right)}^{2}\sigma_{N}{}^{2}+\sigma_{N}{}^{2}\;.$$

The common ratio of *C*^2^ is between 0 and 1. Therefore, as *n* goes to infinity,

$$E\left[x^{2}\right]\equiv \underset{n\to \infty }{\text{lim}}E\left[x_{n}{}^{2}\right]=\left(\frac{{\left(C-1\right)}^{2}}{1-C^{2}}+1\right)\sigma_{N}{}^{2}=\frac{2-2C}{1-C^{2}}\sigma_{N}{}^{2}=\frac{2}{\left(1+C\right)}\sigma_{N}{}^{2}.$$

Therefore, using Eq 13,

$$\text{var}[x]=\sigma_{x}{}^{2}=E[x^{2}]-{\left(E[x]\right)}^{2}=E[x^{2}]=\frac{2}{\left(1+C\right)}\sigma_{N}{}^{2}.$$ (15)

Assuming large enough *n*, the denominator of Eq 12 approximately becomes the variance of *x*, or *σ*_*x*_^2^ multiplied by (*n–* 1), or

$$\sum_{i=1}^{n-1}x_{i}{}^{2}≅(n-1)\sigma_{x}{}^{2}=\frac{2(n-1)\sigma_{N}{}^{2}}{\left(1+C\right)}.$$ (16)

The variance of *x* can also be simply obtained as a stable solution of

$$E[x_{i+1}{}^{2}]=E[{\left(Cx_{i}+N_{i+1}-N_{i}\right)}^{2}]=E[x_{i}{}^{2}],$$

or

$$E[C^{2}x_{i}{}^{2}+N_{i+1}{}^{2}+N_{i}{}^{2}+2Cx_{i}N_{i+1}-2N_{i+1}N_{i}-2Cx_{i}N_{i}]=E[x_{i}{}^{2}].$$ (17)

From Eq 11, *x*_*i*_ is a weighted sum of *N*_1_, *N*_2_, …, and *N*_*i*_. Therefore, using Eq 14, the terms with *N*_*i+*1_ *N*_*i*_ and *x*_*i*_ *N*_*i+*1_ do not contribute to the expectation *E*[.] in Eq 17. In addition, as only *N*_*i*_^2^ in the *x*_*i*_ *N*_*i*_ contributes to the expectation, Eq 17 becomes

$$E[C^{2}x_{i}{}^{2}+N_{i+1}{}^{2}+N_{i}{}^{2}-2CN_{i}{}^{2}]=E[x_{i}{}^{2}].$$ (18)

By definition, the expectation of $N_{i}{}^{2}$ is *σ*_*N*_^2^. Therefore, the expectation of *x*^2^, or *σ*_*x*_^2^ satisfies

$$C^{2}\sigma_{x}{}^{2}+2\sigma_{N}{}^{2}-2C\sigma_{N}{}^{2}=\sigma_{x}{}^{2},$$

or

$$\sigma_{x}{}^{2}=\frac{2-2C}{1-C^{2}}\sigma_{N}{}^{2}=\frac{2}{1+C}\sigma_{N}{}^{2},$$

which is same as Eq 16. Then, the expectation of *C*_*YW*_ becomes

$$E\left[C_{YW}\right]=E\left[\frac{\sum_{i=1}^{n-1}x_{i}x_{i+1}}{\sum_{i=1}^{n-1}x_{i}{}^{2}}\right]≅\frac{E\left[\sum_{i=1}^{n-1}x_{i}x_{i+1}\right]}{\left(\frac{2(n-1)\sigma_{N}{}^{2}}{1+C}\right)}$$ (19)

The numerator of Eq 19 becomes

$$E\left[\sum_{i=1}^{n-1}x_{i}x_{i+1}\right]=E\left[x_{1}x_{2}+x_{2}x_{3}+x_{3}x_{4}+\cdots x_{n-1}x_{n}\right].$$ (20)

From Eq 11,

$$\begin{array}{ll} x_{1}x_{2} & =(C-1)N_{1}{}^{2}+N_{1}N_{2}\\ x_{2}x_{3} & ={\left(C-1\right)}^{2}CN_{1}{}^{2}+(C-1)N_{2}{}^{2}+aN_{1}N_{2}+bN_{2}N_{3}+cN_{1}N_{3}\\ \vdots \; & \ddots \\ x_{n-1}x_{n} & ={\left(C-1\right)}^{2}C^{2n-5}N_{1}{}^{2}+{\left(C-1\right)}^{2}C^{2n-7}N_{2}{}^{2}+\cdots +(C-1)N_{n-1}{}^{2}+\text{terms with }N_{p}N_{q}\text{ (}p\neq q). \end{array}$$

By Eq 14, only terms with *N*_*i*_^2^ contribute to the expectation *E*[.] in Eq 20, whereas terms with *N*_*p*_ *N*_*q*_ (*p* ≠ *q*) do not contribute to the expectation. Therefore,

$$\begin{array}{ll} E\left[\sum_{i=1}^{n-1}x_{i}x_{i+1}\right] & =E\left[(C-1)N_{1}{}^{2}\right]\\ & +E\left[{\left(C-1\right)}^{2}CN_{1}{}^{2}+(C-1)N_{2}{}^{2}\right]\\ & \vdots \;\ddots \\ & +E\left[{\left(C-1\right)}^{2}C^{2n-5}N_{1}{}^{2}+{\left(C-k\right)}^{2}C^{2n-7}N_{2}{}^{2}+\cdots +(C-1)N_{n-1}{}^{2}\right] \end{array}$$ (21)

By definition of *N*_*i*_, the expectation of $N_{i}{}^{2}$ is *σ*_*N*_^2^. Therefore, Eq 21 becomes

$$\begin{array}{ll} E\left[\sum_{i=1}^{n-1}x_{i}x_{i+1}\right] & =\{(C-1)\\ & +{\left(C-1\right)}^{2}C+(C-1)\\ & +{\left(C-1\right)}^{2}C^{3}+{\left(C-1\right)}^{2}C+(C-1)\\ & \;\vdots \;\ddots \\ & +{\left(C-1\right)}^{2}C^{2n-5}+{\left(C-1\right)}^{2}C^{2n-7}+\cdots +(C-1)\}\times \sigma_{N}{}^{2} \end{array}$$ (22)

Eq 22 can be re-written as

$$\begin{array}{ll} \frac{E\left[\sum_{i=1}^{n-1}x_{i}x_{i+1}\right]}{\sigma_{N}{}^{2}} & =(C-1)(n-1)\\ & +{\left(C-1\right)}^{2}C\\ & +{\left(C-1\right)}^{2}C^{3}\;+\;{\left(C-1\right)}^{2}C\\ & +{\left(C-1\right)}^{2}C^{5}\;+\;{\left(C-1\right)}^{2}C^{3}\;+\;{\left(C-1\right)}^{2}C\\ & \;\vdots \;\ddots \\ & +{\left(C-1\right)}^{2}C^{2n-5}+{\left(C-1\right)}^{2}C^{2n-7}+\;\cdots \;+{\left(C-1\right)}^{2}C \end{array}$$ (23)

The sum of the geometric sequence becomes

$$C+C^{3}+C^{5}+\cdots C^{2m-5}=\frac{1-C^{2(m-2)}}{1-C^{2}}C.$$

Therefore, Eq 23 becomes

$$\begin{array}{ll} \frac{E\left[\sum_{i=1}^{n-1}x_{i}x_{i+1}\right]}{\sigma_{N}{}^{2}} & =(C-1)(n-1)+{\left(C-1\right)}^{2}\left\{\frac{1-C^{2(n-2)}}{1-C^{2}}C+\frac{1-C^{2(n-3)}}{1-C^{2}}C+\cdots +C\right\}\\ & =(C-1)(n-1)+{\left(C-1\right)}^{2}\frac{C}{1-C^{2}}\left\{(n-2)-\left(C^{2(n-2)}+C^{2(n-3)}+\cdots +C^{2}\right)\right\}\\ & =(C-1)(n-1)+{\left(C-1\right)}^{2}\frac{C}{1-C^{2}}\left\{(n-2)-\frac{C^{2}}{1-C^{2}}\left(1-C^{2(n-2)}\right)\right\} \end{array}$$ (24)

From Eqs 19 and 24,

$$\begin{array}{l} E\left[C_{YW}\right]≅\frac{E\left[\sum_{i=1}^{n-1}x_{i}x_{i+1}\right]}{\left(\frac{2(n-1)\sigma_{N}{}^{2}}{1+C}\right)}\\ =\frac{\left(C^{2}-1\right)}{2}+\frac{(1-C)C}{2(n-1)}\left\{\left(n-2)-\frac{C^{2}}{1-C^{2}}(1-C^{2(n-2)}\right)\right\} \end{array}$$ (25)

This expression shows that the bias is hardly reduced, even when *n* is large. Assuming large *n*,

$$E\left[C_{YW}\right]≅\frac{C-1}{2}.$$ (26)

Note that the expectation of the estimate of *C* by the Yule-Walker method for large *n* is negative because 0 < *C* < 1. This means that the estimation is severely biased, if we use the Yule-Walker algorithm to measure the correction gain of the learning model. For a general autoregressive (AR) process, the Yule-Walker equation shows a finite bias [41, 42], but not to the same extent. The substantial bias in this case originated from the anti-correlated noise added to the linear relation between *x*_*i*_ and *x*_*i*+1_. This contrasts with the added noise in the general AR process that has no correlation.

### Equation for the Improved Estimation

From Eq 25, for large enough *n*, the expectation of C can be written as

$$E\left[C_{YW}\right]≅\frac{\left(C^{2}-1\right)}{2}+\frac{(1-C)C}{2(n-1)}(n-2),$$

or

$$C^{2}+(n-2)C-(n-1)(1+2E\left[C_{YW}\right])≅0.$$ (27)

From Eq 26, the term 1 + 2*E*[*C_YW_*] is positive. Therefore, the quadratic equation (Eq 27) has one positive root and one negative root. We are interested in the positive root, and the coefficient *C* is estimated as

$$C≅\frac{-(n-2)+\sqrt{{\left(n-2\right)}^{2}+4(n-1)(1+2C_{YW})}}{2}.$$ (28)

Accordingly, the true correction gain *B* can be approximated as

$$B≅\frac{n-\sqrt{{\left(n-2\right)}^{2}+4(n-1)(1+2C_{YW})}}{2}.$$ (29)

Simulation confirmed that Eq 29, the Adjusted Yule Walker or AYW method improves the estimate. Fig 3 and Table 1 show that the bias was significantly reduced by the AYW method to less than 5% as *n* increased. This improvement was neither affected by the type of the noise distribution, irrespective of whether the noise came from a normal, a uniform, or an asymmetric lognormal distribution. It was also unaffected by the magnitude of the noise level *σ*_*N*_.

If the actual noise variance is of interest, the time series of *N*_*i*_ can also be obtained in the following manner. The motor output *x*_*i*_ is directly measured. After *C* is identified from Eq 28, the time series of (*N*_*i*+1_ –*N*_*i*_) can be computed from Eq 10. With an arbitrarily assumed value of *N*_1_, *N*_*i*_ can be obtained. Then, *N*_1_ and *N*_*i*_ can be recomputed so that *N*_*i*_ has zero mean.

## Discussion

A variety of studies on error-based adaptation and learning in computational neuroscience have regarded the correction gains of the learning process as an essential piece of information. These correction gains, feedback gains, or learning rates have been typically identified by least-square curve fitting, frequently using the widely available Levenberg-Marquardt algorithm. These methods were based on the premise that the effect of noise averages out by the regression. Indeed, the least-square curve fitting provides a reasonably accurate measure of the correction gain when the performance changes are considerably higher than the amplitude of the noise processes. However, later in learning when performance approaches a plateau, we showed that the noise in the system induces a substantial bias in the estimate of the correction gain, when relying on conventional least-square regression. Using a simple autoregressive learning model, we then devised an improved method that quantifies and corrects the bias.

### Necessity of Improved Estimation Method

Our simulation results demonstrate that the conventional methods based on regression always overestimate the correction gain. The noise induces uncertainty (i.e., non-zero variance in the estimation) as well as a clear bias in the mean or expected value. As shown in Fig 3 and Table 1, the bias can be substantial, even in the simplest case of a learning model. Furthermore, the bias was not reduced by increasing the trial number *n* in the estimates by the Levenberg-Marquardt least-square algorithm. The subsequent analytical derivation proved that the bias from a linear regression method, such as the Yule-Walker equation, converges to a non-zero value as *n* approaches infinity, as shown in Eq 26. The same behavior is the likely cause for the observed bias in the Levenberg-Marquardt estimates, evidenced in our simulations. This robustness of the bias against the data size highlights the serious limitation of the conventional estimation of the correction gain.

A possible alternative to the least-square regression methods is the Expectation Maximization (EM) algorithm that has been used to identify parameters of a stochastic model from the observed data [43, 44]. The EM algorithm can reduce the bias due to noise with long enough time series, but it cannot estimate model parameters that are assumed to be deterministic [33]. Consequently, for the simple iterative learning process as in Eq 7, which is a kind of Autoregressive Moving Average (ARMA) model, the parameter *B* cannot be estimated by the EM algorithm [33]. Hence, other methods need to be identified.

### Insensitivity of Bias to Noise Magnitude and Distribution

The results show that the bias due to conventional estimates does not depend on the magnitude of the noise in the data. This robustness of the bias against the noise magnitude further underlines the importance of the bias reduction, suggesting that the bias needs to be adjusted, even if the noise magnitude is relatively low.

Another important observation is that the bias is insensitive to the noise distribution. Most of mathematical learning models have typically assumed white Gaussian noise, which is mathematically well-defined and convenient to analyze. However, several studies convincingly suggest that the noise distribution in biological systems is closer to an asymmetric lognormal distribution rather than a Gaussian distribution [45–48]. The insensitivity of bias to the noise distribution enables the AYW method to provide an improved estimation without relying on the common but vulnerable assumption of most learning models.

As the bias is induced by noise, it may appear necessary to identify the noise properties in the data set to assess and eliminate the bias. However, our analytical analysis shows that we do not need detailed knowledge of the noise properties to estimate the bias. Eq 25 demonstrates that the bias does not depend on the noise level *σ*_*N*_. In addition, we did not assume any specific distribution of the noise to derive the closed form of the bias; we only assumed that the newly added noise in the current trial is independent of the noise in the previous trial. This implies that we can apply the same method for a variety of systems with different levels and distributions of noise. For example, we can use the same AYW method to estimate the correction gain of young and healthy subjects or patients who may have different types and degrees of noise.

### Considerations and Limitations

While the noise distribution is of no concern, our analysis assumes that the noise samples added to each trial have no correlation. While the assumption of uncorrelated noise in human movement has been generally accepted in learning models and is supported by some experimental observations [45], other studies have also shown significant correlations, positive and negative, in the variability of the motor output. For example, time series of stride intervals in human walking and heart beats exhibit long-range correlations, suggesting that the variability in those motor behaviors cannot be modeled as white noise [49, 50]. Hence, caution is necessary when applying the AYW method to a system with correlated or anti-correlated noise.

However, we need to distinguish the variability of the observed motor output from the assumed noise that causes the variability. In Eq 7, the variability of *x* may exhibit correlation, even if *N* has no correlation. Actually, a recent study showed that the long-range correlations in stride intervals of human walking and other rhythmic motor behavior may be explained by uncorrelated noise filtered by stable rhythmic dynamics of the sensorimotor system [51]. The assumption that the original unfiltered source of variability is white noise is also common and highly effective in control and signal processing theory as well as in computational biology. It is also the basis of the widely-applicable Kalman filter [52]. Hence, the observed correlated variability in the motor output does not invalidate the AYW method, which assumes uncorrelated noise.

The AYW method significantly reduces the bias when the length of the time series is sufficiently long (*n* ≥ 400). However, the accuracy of the AYW method is relatively low when the length of the time series is short (*n* ≤ 50), although the bias is always smaller than the bias in the least-square method (see Table 1). This inaccuracy with insufficient data is due to the fact that the bias reduction by the AYW method depends on Eq 16, which assumes large *n*. Future work may resolve this inaccuracy when only insufficient data are available.

While the AYW method provides a less biased estimation, it shows higher variability than the least-square method, particularly when the size of the data is small. This high variability may limit the efficacy of the AYW method when a distinction between correction gains of two groups is required and only a small data set is available. This trade-off between bias and variance has already been identified in other estimation methods and discussed in prior studies [53, 54]. Development of a new method that optimizes the efficacy considering the trade-off between bias and variance is deferred to future work.

Like many other studies, our approach assumed that the correction gain or the learning rate is constant. This assumption, though widely used, may not be strictly accurate; the learning rate is approximately constant for small errors, but may change when the errors become larger [55, 56]. This study addresses the bias of the correction gain estimation when the learning process approaches skilled levels or steady state, where small errors are prevalent. Therefore, the assumption of a constant correction gain is sufficiently valid within the scope of this study.

For initial study, our analysis used a highly simplified one-dimensional model of motor learning, and therefore, generalization to other models and multi-dimensional cases will require further considerations. For example, for rapid pointing movements, van Beers showed that this simple model was insufficient to account for the observed structure of variability [13]. However, our chosen model contains the essential components of motor learning: feedback through error information and unplanned variability due to noise. This basic simple learning model was used to clearly demonstrate the influence of noise on estimation.

### A Representative Problem for the Influence of Noise on Data Analysis

The observed bias is an example for how the dynamics of noise can introduce significant distortions in the analysis. It is widely assumed that noise is “neutral” if it has zero mean, as the effect of noise averages out with a large amount of data. However, the accumulated effect of noise may not always remain neutral, if the noise is processed by the analysis, such as in the conventional least-square curve fitting. Another non-intuitive effect of noise on estimation was highlighted in a recent study on Floquet multipliers, a method for assessing orbital stability of rhythmic movements. Noise induces a bias in the estimation of orbital stability [57]. Another example for the surprising effect of noise is the two-thirds power law that is widely observed in human movements [58–61]. It has been shown that this subtle relation between velocity and curvature can be generated by Gaussian noise alone [62]. Hence, experimental assessment and interpretation of the power law as a fundamental principle of movement generation needs caution.

Noise is ubiquitous and deserves more attention as its dynamics may influence the estimation of the “deterministic” elements of the system. Our study is only one example to show that the common belief that the accumulated effect of noise is neutral needs revision. More work is needed to examine more complex learning models and the estimation of noise processes from the data.
